# Automated Segmentation and Measurements of Pulmonary Cysts in Lymphangioleiomyomatosis across Multiple CT Scanner Platforms over a Period of Two Decades

**DOI:** 10.3390/bioengineering10111255

**Published:** 2023-10-27

**Authors:** Simone Lee, Alfredo Lebron, Brianna Matthew, Joel Moss, Han Wen

**Affiliations:** 1 Biochemistry and Biophysics Center, National Heart, Lung, and Blood Institute, National Institutes of Health, Bethesda, MD 20892, USA; 2 Pulmonary Branch, National Heart, Lung, and Blood Institute, National Institutes of Health, Bethesda, MD 20892, USA

**Keywords:** LAM, CT, cyst, automated segmentation, image analysis

## Abstract

(1) Background: Lymphangioleiomyomatosis is a genetic disease that affects mostly women of childbearing age. In the lungs, it manifests as the progressive formation of air-filled cysts and is associated with a decline in lung function. With a median survival of 29 years after the onset of symptoms, computed-tomographic monitoring of cystic changes in the lungs is a key part of the management of the disease. However, the current standard method to measure cyst burdens from CT is semi-automatic and requires manual adjustments from trained operators to obtain consistent results due to variabilities in CT technology and imaging conditions over the long course of the disease. This can be impractical for longitudinal studies involving large numbers of scans and is susceptible to subjective biases. (2) Methods: We developed an automated method of pulmonary cyst segmentation for chest CT images incorporating novel graphics processing algorithms. We assessed its performance against the gold-standard semi-automated method performed by experienced operators who were blinded to the results of the automated method. (3) Results: the automated method had the same consistency over time as the gold-standard method, but its cyst scores were more strongly correlated with concurrent pulmonary function results from the physiology laboratory than those of the gold-standard method. (4) Conclusions: The automated cyst segmentation is a competent replacement for the gold-standard semi-automated process. It is a solution for saving time and labor in clinical studies of lymphangioleiomyomatosis that may involve large numbers of chest CT scans from diverse scanner platforms and protocols.

## 1. Introduction

**Figure 1:**
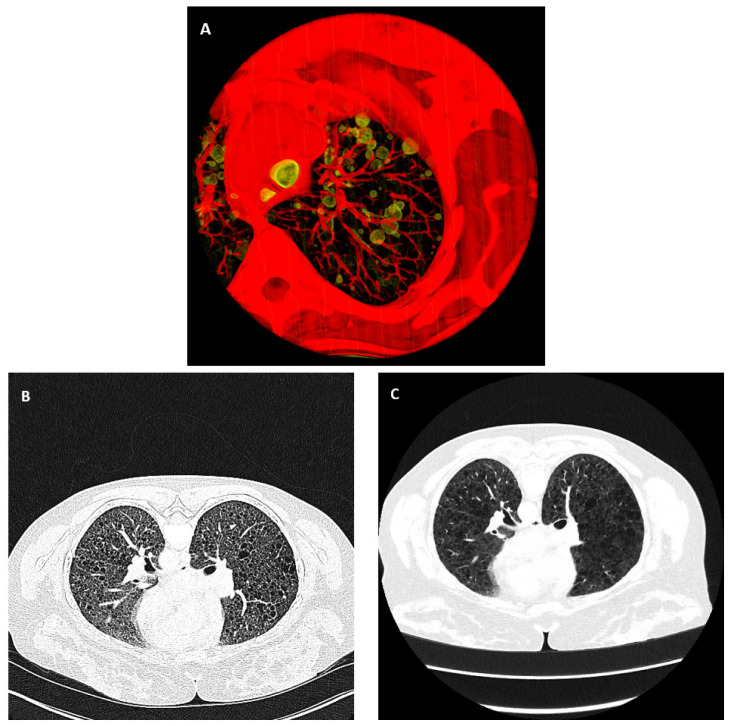
Air-filled pulmonary cysts from the disease lymphangioleiomyomatosis and the variability of chest CT images over time and across different scanner platforms. (**A**) A three-dimensional rendered view of a portion of the lung from the chest CT scan of a patient. The bubble-like structures in the lung highlighted in yellow are air-filled cysts, a hallmark of the disease. (**B**,**C**) High-resolution CT images of the same location in the chest of the same patient were acquired 12 years apart from two different scanner platforms. Both are displayed in the grayscale range of −1100 HU to 0 HU. This is an example of the variability of image quality and characteristics.

Lymphangioleiomyomatosis (LAM) is a genetic multi-system disease that causes the progressive formation of air-filled cysts in the lungs (Figure 1A), together with the loss of lung function and occurrences of pneumothorax (the collapse of a lung) and chylothorax (the buildup of fluid in the chest) [1,2,3,4,5,6,7]. The disease primarily affects women of childbearing age [1,2,3,4,5,6,7]. There are two types of LAM disease: a sporadic form, which occurs in approximately 3 to 8 per million women, and an inherited form associated with the rare genetic disease of tuberous sclerosis complex (TSC) [6]. With treatment and supplemental oxygen, patients have a transplant-free median survival of 29 years after the onset of symptoms [6,8,9]. Computed tomographic (CT) measurements of the cystic changes in the lungs over time are an integral part of the management of the disease, providing valuable markers of the severity and rate of disease progression and aiding treatment decisions [10,11,12,13,14,15,16,17,18].

In LAM, the extent of pulmonary cysts correlates with the decline of lung function [10,11,12,13,14,15,16,17,18,19,20]. CT-derived quantitative assessment of pulmonary cysts initially was a visual determination of the percentage of the lung that involved cysts [19,20]. Later, semi-automated software was introduced to provide measures of cystic changes from high-resolution chest CT (HRCT) scans, including the total volume of the pulmonary cysts normalized to the total parenchyma or air volume in the lungs [10,11,12,13,14,15,16,17,18]. Currently, commercial semi-automated software dedicated to pulmonary cyst measurements is available on some scanner platforms (Canon Medical Systems USA, Inc., Tustin, CA, USA), which provides the volume percentage of the lungs occupied by cysts, or “cyst score” [10].

Given the long duration of the disease, it is challenging to obtain consistent and accurate measurements of the cystic changes in the lungs for individual patients over a period of decades due to changes in scanner platforms, imaging technologies, and physiological conditions. As a result, current FDA-approved gold-standard methods for the identification and measurement of cysts in CT scans include manual adjustments and corrections by trained operators [14,15,16,21,22]. However, when it comes to longitudinal studies involving a large number of CT scans over several decades from a population of patients, the time needed to perform such semi-automated analysis would be impractical, and the results are also susceptible to inter-operator variability and intra-operator drifts. For such studies, fully automated analysis is desirable.

Previously, an automated method has been described to obtain the global volume percentage of cysts in a group of patients with identical scan and image reconstruction settings on a single scanner platform [18]. However, this method does not identify the pulmonary cysts in the images, thus precluding measurements such as the size and the spatial distribution of the cysts and tissue texture analysis around the cysts, which are clinically relevant [14,15,22,23,24]. It is also based on assumptions about the global histogram distribution of radiodensity values in the lungs, which could not be applied to studies that involve multiple scanner platforms and scan/reconstruction settings.

Therefore, there is an unmet need for automated segmentation and measurements of pulmonary cysts in HRCT images of LAM disease, particularly in the presence of instrumental and physiological variabilities. In this paper, we introduce an automated method to meet this need. We tested its longitudinal consistency and accuracy against the FDA-approved gold-standard semi-automated software in a longitudinal study involving multiple scanner platforms over a period of two decades.

### 1.1. Variability of the Scanner Platform and Imaging Conditions in Longitudinal Studies

To describe the level of variability in HRCT scans that we encountered in longitudinal studies, in a survey of 268 high-resolution chest CT (HRCT) image series from 24 LAM patients over a period of 23 years, we found them to be from 12 different scanner models from four different manufacturers (Ge Medical Systems LightSpeed Ultra, Ge Medical Systems LightSpeed QX/i, Ge Medical Systems HiSpeed CT/I, Ge Medical Systems Genesis_Hispeed_RP, Siemens Definition, Siemens Biograph128, Siemens Somatom Force, Siemens Somatom Definition Flash, Philips Brilliance 64, Philips Mx8000 IDT 16, Toshiba Aquilion One, and Canon Aquilion One/Prism). Eighteen different image reconstruction kernels were used at various times over the two decades. Of these image series, 39% were performed with contrast agents, with varying delays after contrast injection and levels of contrast enhancement of the lung tissue. An example of the diverse image quality and appearance is illustrated in Figure 1B,C. The two images were from the same patient. They were acquired on two different scanner models, 12 years apart.

### 1.2. Variability of the Radiodensity of Air Inside and Outside the Chest

**Figure 2:**
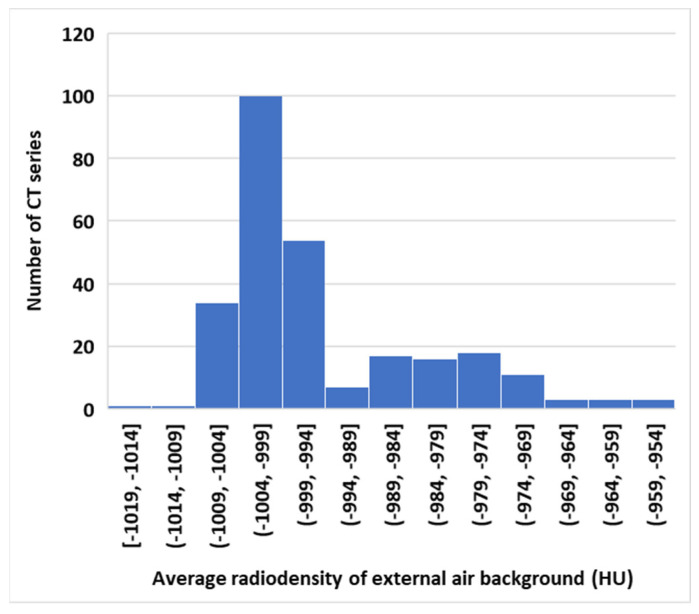
Frequency histogram of the average radiodensity of the empty air background around the body of 268 chest CT image series of LAM patients. The scans were from 12 different scanner models over a period of 23 years. The air background, by definition, should have a radiodensity of −1000 HU. The actual values were diverse due to variable scanner technology and the quality of scanner calibration.

**Figure 3:**
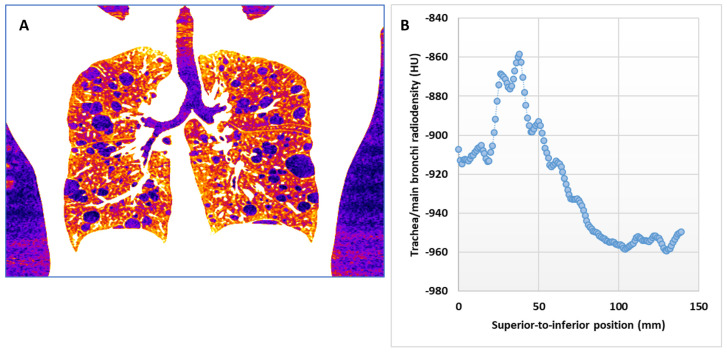
Variability of the radiodensity of air in large airways along the superior–inferior direction. (**A**) A reformatted coronal section through an HRCT image series. The radiodensity of air in the trachea is seen to vary with the axial position. The radiodensity of air in the pulmonary cysts in the apical region of the lungs followed the same variation. (**B**) A plot of the average radiodensity of air in the trachea and the main bronchi as a function of the position in the superior–inferior direction.

As a result of the diverse scanner platforms and imaging conditions summarized above, one aspect of the image variability relates to the radiodensity of large air spaces, both internal and external to the lungs. First, the average radiodensity of the external air background in an image series, which ideally should be calibrated to −1000 Hounsfield units (HU), ranged from −954 HU to −1018 HU. The frequency histogram of the air background level among the image series is shown in Figure 2, with a mean ± standard deviation of −994.6 ± 11.6 HU. Secondly, the radiodensity of large internal air spaces in the lungs, including the trachea and the main bronchi, may be dependent on their position in the superior–inferior direction, especially in the apical region of the chest. This is visible in the coronal cross-section through an HRCT series (Figure 3A). This is also confirmed in the plot of the radiodensity in the trachea and the main bronchi versus the vertical position (Figure 3B). Since pulmonary cysts are a type of air space within the lungs, their radiodensity will fluctuate along with both the external air background and the internal large airways.

### 1.3. Variability of the Radiodensity of the Pulmonary Parenchyma

**Figure 4:**
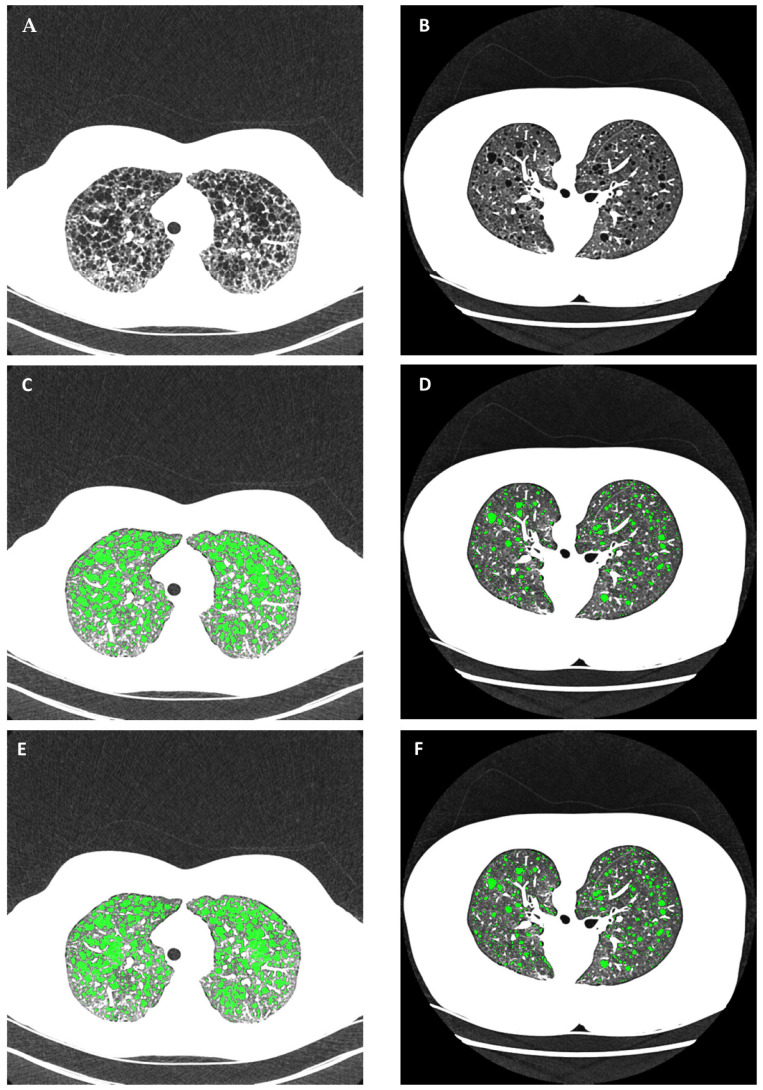
An example of the variability in the radiodensity of air and non-cystic lung parenchyma across different patients and scanner platforms and the consequences on cyst segmentation. (**A**,**B**) Chest HRCT scans of two patients from two different scanner platforms in supine and prone positions, respectively. Both are displayed in the grayscale range of −1050 HU to −600 HU to highlight lung parenchymal structures. The average radiodensity of the air background outside the body was −971 HU in (**A**) and −1006 HU in (**B**). The average radiodensity of non-cystic lung parenchyma was −805 HU in (**A**) and −918 HU in (**B**). (**C**,**D**) Optimal cyst segmentation was determined by a trained operator using the gold-standard commercial semi-automated software. The threshold radiodensity for cystic areas was adjusted by the operator to −880 HU for the image series in (**A**) and −970 HU for the image series in (**B**). The cystic areas are highlighted in green. (**E**,**F**) Automated cyst segmentation without user input. The automated method accounted for the variability of radiodensity values to provide the appropriate segmentation (highlighted in green).

**Figure 5:**
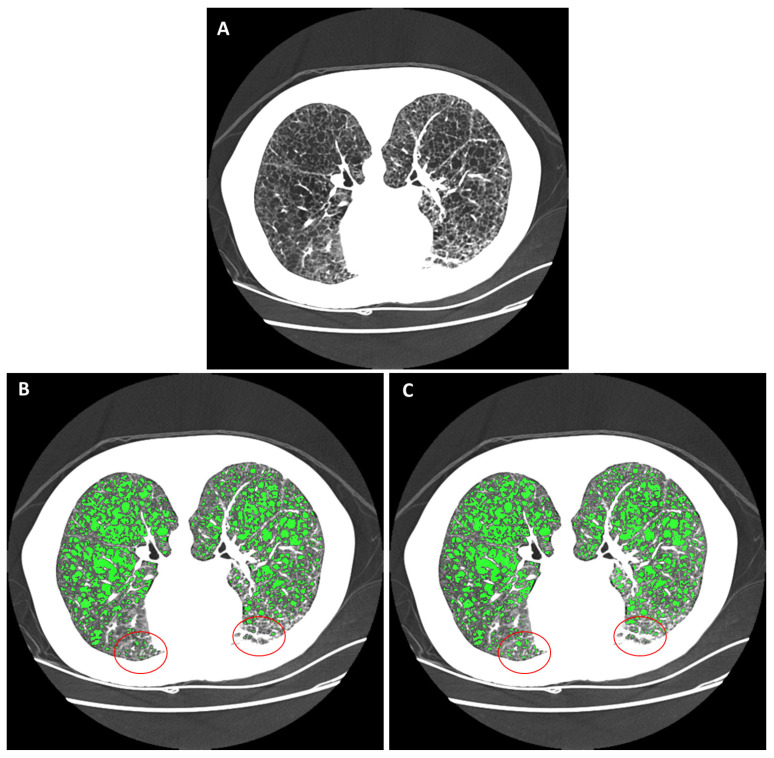
An example of within-section variability of the radiodensity of the pulmonary parenchyma and its impact on cyst segmentation. (**A**) An HRCT section of a LAM patient lying in the prone orientation is displayed in the grayscale range of −1100 HU to −500 HU. The anterior portion of the lungs was enhanced due to positional atelectasis. (**B**) The gold-standard semi-automated segmentation by a trained operator, who set a global threshold for cystic areas to −930 HU. Cystic areas are highlighted in green. The anterior region of the lungs was under-segmented due to the elevated parenchymal radiodensity there (highlighted by the red circles). (**C**) The automated cyst segmentation. The automated method accounted for regional variations of radiodensity, which mitigated the problem of under-segmentation in the anterior region.

Another aspect of the image variability relates to the radiodensity of the pulmonary parenchyma. These values varied from patient to patient (Figure 4) and occasionally within the same section of a scan (Figure 5). Physiologically, aside from inherent variability among a population, the parenchymal radiodensity is influenced by the level of compliance on the part of the patient during an inspiratory scan, as well as transient radiologic abnormalities such as atelectasis that may be present in local areas of the lung. In the example of Figure 4, the mean parenchymal radiodensity of the two patients differed by 113 HU, or 120% of the apparent density of the pulmonary parenchyma of the second patient (Figure 4A,B). As a result, the manually adjusted thresholds in the semi-automated segmentation of cysts differed by 90 HU between the two patients, at −880 HU and −970 HU, respectively (Figure 4C,D). In the example of Figure 5, atelectasis in the anterior portion of the lungs leads to elevated radiodensity in that area. Consequently, in the semi-automated cyst segmentation, the optimal setting determined by the operator under-segmented the cysts in the anterior region.

In light of the CT variabilities described above, we developed an automated segmentation of pulmonary cysts with radiodensity thresholds that were adapted to the local radiodensity of non-cystic parenchyma, which in turn was calculated with a smoothed histogram filter algorithm [25,26]. In the media industry, smoothed histogram filters are commonly used to produce stylized graphics [25]. We used it previously with satisfactory results in automated segmentation of pulmonary blood vessels in post-COVID-19 patients [26].

## 2. Materials and Methods

### 2.1. Study Population

We performed a retrospective longitudinal study of 192 chest CT image series from 24 LAM patients enrolled in the clinical research protocol “Role of Genetic Factors in the Pathogenesis of Lung Disease” (clinicaltrials.gov NCT00001532) at the Clinical Center of the National Institutes of Health, Bethesda, Maryland, USA, over a period of 23 years from 1999 to 2022. The protocol was approved by the National Heart, Lung, and Blood Institute, National Institutes of Health Institutional Review Board (IRB # 96-H-0100). The patients were monitored with chest scans of various forms at intervals of 1 to 2 years during this period. All image series included in this study received the standard semi-automated cyst measurements by FDA-approved commercial software. A subset of the CT examinations had concurrent pulmonary function tests (PFTs) in the pulmonary physiology laboratory during the same hospital visit. The authors had access to information that could identify individual participants during or after data collection. All patients were female and had been diagnosed with LAM according to the American Thoracic Society/Japanese Respiratory Society criteria [27,28].

### 2.2. Metrics for Assessing the Quality of the Cyst Segmentation

**Figure 6:**
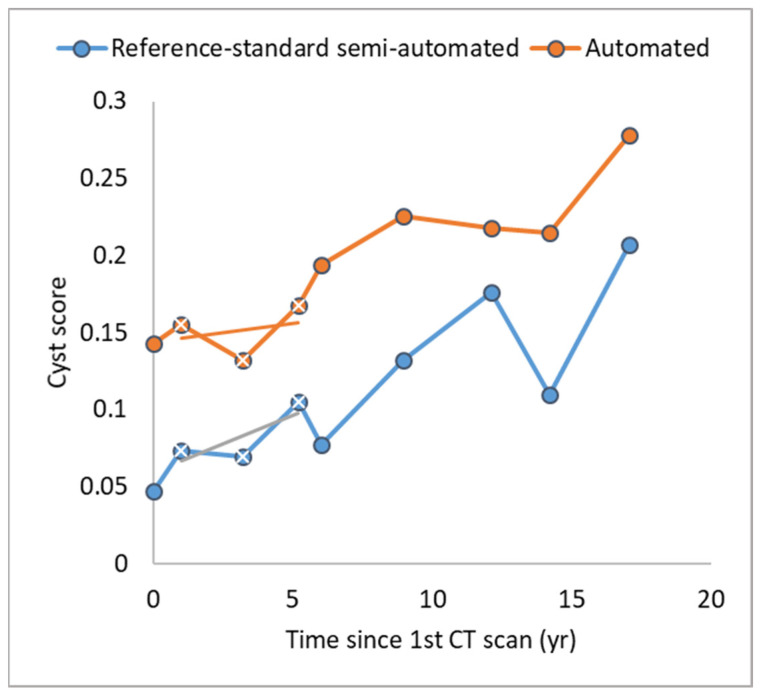
Illustration of the measurement of inconsistencies in the cyst scores over time for individual patients (longitudinal inconsistency). The graph plots the cyst scores of a LAM patient over a period of 18 years, during which she received 9 CT examinations. The scores from the two segmentation methods are plotted in separate lines. The rapid fluctuation of the scores from exam to exam reflects measurement inconsistencies due to changes in instrumental factors and imaging conditions. The longitudinal inconsistency is defined for each group of 3 consecutive scores as the standard error of the linear regression over the three data points. In the graph, the measure represents the deviations of the three consecutive data points from the short straight line. There are 7 measurements of longitudinal inconsistency in total for the time course in this example.

In LAM, conventional measures of the extent of cystic changes in the lungs are based on the percentage of the total lung volume replaced by cysts, called the cyst score [14,17,18,19,20]. The quality of the cyst segmentation was evaluated through the cyst score and two quantitative metrics. The first is the longitudinal inconsistency over time, illustrated in Figure 6. In the graph of the cyst scores vs. time for an individual patient, variabilities of scan technology and physiological conditions can lead to rapid fluctuations of the values from year to year or inconsistency of the measurement over time. The metric of longitudinal inconsistency is defined as the standard error of linear regression for every group of three consecutive cyst scores (Figure 6). Thus, a patient who underwent 9 CT scans over a period of 18 years will have 7 measures of longitudinal inconsistency. We additionally analyzed the maximum of the inconsistency measures as the worst inconsistency for each patient.

The second metric evaluates the accuracy of the cyst scores by their correlation with concurrent pulmonary function tests [18]. The pulmonary function tests included the spirometry tests of forced expiratory volume in the first second as a % of predicted reference values (FEV1_pp), FEV1 normalized to the vital capacity as a % of predicted reference values (FEV1/FVC_pp), and the laboratory test of the diffusion capacity of the lungs for carbon monoxide adjusted for hemoglobin as a % of predicted reference (DLco_adj_pp) [29]. These correlations were calculated for all CT scans where the patients had concurrent PFTs on the same hospital visit.

### 2.3. Reference Gold-Standard Semi-Automated Cyst Segmentation

The reference standard against which the new automated analysis was evaluated was FDA-approved commercial software on a modern scanner platform (Canon Medical Systems USA, Inc., Tustin, CA, USA). The analysis was performed by two operators with 1 and 2 years of experience in scoring HRCT scans of LAM patients. They were blinded to the results of the automated method.

The analysis consisted of a sequence of steps. After importing the image DICOM series into the software, it first performed a fully automated segmentation of the lung volume and the large airways. The optimal segmentation of the cysts was then determined visually by an operator through manual adjustment of a global threshold of radiodensity. Manual editing of individual areas was available if needed. Examples of cyst segmentation by the reference standard software are illustrated in Figure 4C,D and Figure 5B. Following the segmentation, the total volume of cysts was calculated, and the ratio of the total cystic volume over the total lung volume provided the cyst score.

### 2.4. Automated Cyst Segmentation

**Figure 7:**
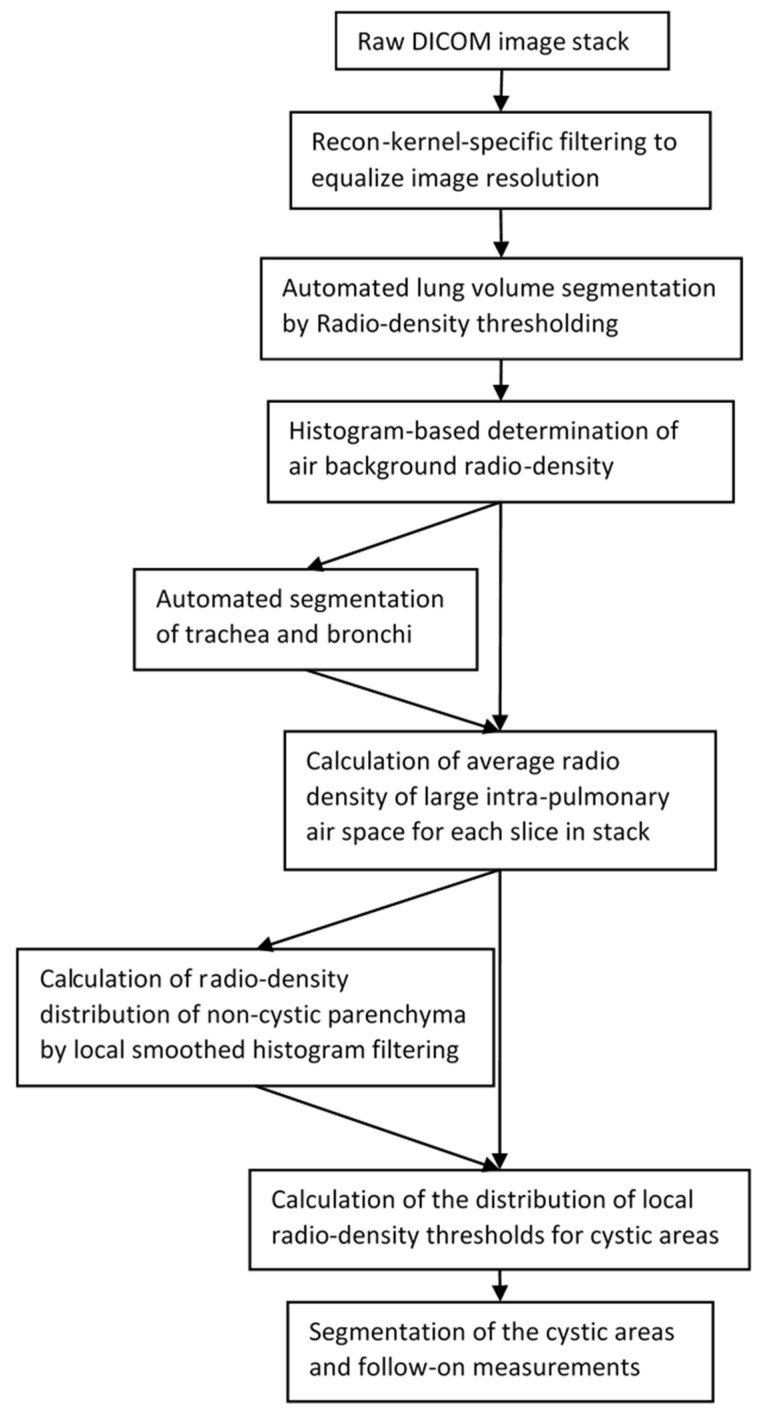
Diagram of the pipeline of the automated method to identify and segment the cystic areas in high-resolution chest CT (HRCT) series of patients with lymphangioleiomyomatosis (LAM).

The pipeline of the automated analysis included a sequence of modules (Figure 7). The basic idea was to determine the cystic areas according to local radiodensity thresholds that were set at the midpoint between the local values of large air voids and non-cystic parenchyma. These values were calculated from a combination of automated segmentation of large airways and local smoothed histogram filtering. An advantage of smoothed histogram filtering is that the mode (peak) of the histogram is unaffected by small changes in the upper and lower limits of the range of values that were deemed relevant, making it a robust method.

The first module in the pipeline applied a conditional Gaussian filter to the raw image series to obtain an approximate in-plane resolution of 0.529 mm. We found in practice that the resolution and noise level of the raw image series were largely determined by the image reconstruction kernel used. We encountered 18 different reconstruction kernels on the 12 scanner models. Empirically, we compiled a list of the appropriate filter widths for the 18 kernels, which was then applied to all image series accordingly.

The next modules included automated segmentation of the lung volume based on a threshold of radiodensity [26,30], followed by automated segmentation of the trachea in the superior sections of the image series based on radiodensity thresholding. The upper trachea was then automatically extended down to the tree of large airways based on radiodensity thresholding and connectivity. The next step was to determine the average radiodensity of the empty air space around the body. This was based on the frequency histogram of the radiodensity of all voxels in the imaged volume. The air background value was identified as the first substantial peak in the histogram above the value of −1024 HU. It is noted as I_background_.

The next module combined the airway and air background information from the previous module to derive the typical radiodensity of a large air space in the chest, such as a bronchus or a large cyst. It first determined the average radiodensity within the segmented large airways (Figure 3B). This was done on a section-by-section basis for all sections that contained segmented airways. The value at the most inferior position was then copied to all remaining sections below. These values are noted as I_airway_(z), where z is the position of the CT section in the inferior–superior direction. Then, the average of the global air background value and the airway value of each section was taken as the typical radiodensity of intra-lung air space in that section:

I_air_(z) = [I_airway_(z) + I_background_]/2. This value served as a reference point for local smoothed histogram filtering in the next step.

**Figure 8:**
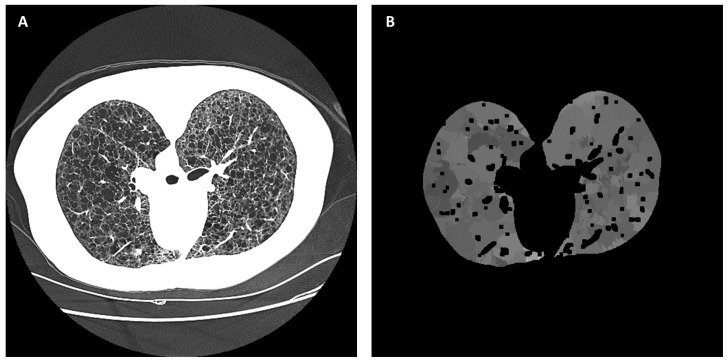
An example of deriving the radiodensity distribution of non-cystic parenchyma in the lungs by a local smoothed histogram filter. (**A**) The raw HRCT section is displayed on a grayscale of −1100 HU to −500 HU. (**B**) The calculated radiodensity distribution of non-cystic parenchyma in the lungs. The dark voids in the distribution are areas of bright vessels.

The next module determined the typical radiodensity of non-cystic parenchyma for all locations in the lungs. In each CT section, a locally smoothed histogram filter was applied to the radiodensity values of all areas in the lungs, excluding the previously segmented large airways and large vessels. The histogram window was a circular area of 35 mm in diameter with a smoothed Gaussian profile that gave more weight to pixels near the center of the window. At each image pixel, a weighted frequency histogram within the radiodensity range of I_air_(z)+25 and −500 HU was calculated from the values of all pixels within the histogram window centered at the pixel. This range of radiodensity excluded large air voids and blood vessels. Then, the radiodensity of the local non-cystic parenchyma, I_p_(x, y, z), was defined as the dominant mode of the local histogram. An example of the raw CT image (Figure 8A) and the calculated image of the radiodensity of non-cystic parenchyma (Figure 8B) are given in Figure 8. It should be noted that the histogram filter would not yield results at the centers of cysts or air voids if they were larger than the histogram window. However, as explained below, this would not affect the identification and segmentation of the cysts.

In the last module of the pipeline, the cysts in the lungs were identified as areas of radiodensity that meet the condition of I(x, y, z) < [I_p_(x, y, z) + I_air_(z)]/2 where I_p_(x, y, z) exists, or I(x, y, z) < I_air_(z) + 25 HU where I_p_(x, y, z) is not available, such as at the centers of very large cysts. The cyst score was then calculated by summing the total volume of the cystic areas and dividing the result by the total lung volume.

## 3. Results

In all statistical results, a *p*-value of less than 0.05 is considered statistically significant. An example comparing automated cyst segmentation with commercial semi-automated segmentation is given in Figure 4. The example included two image series from two patients with markedly different radiodensities of the lung parenchyma (Figure 4A,B). In the gold-standard semi-automated analysis, the operator set the global threshold for cysts to −880 HU and −970 HU, respectively, to attain satisfactory segmentation in each series (Figure 4C,D). The automated method provided appropriate cyst segmentation in both series without user input (Figure 4E,F).

An example of cyst segmentation in the presence of variable parenchymal radiodensity within the same section is given in Figure 5. In this example, the anterior portion of the chest was enhanced by positional atelectasis (Figure 5A). The reference standard semi-automated segmentation was optimized by the operator at a global threshold of −930 HU for cysts (Figure 5B). However, the anterior portion (outlined in red in Figure 5B) was under-segmented. This was mitigated by the automated segmentation (Figure 5C).

### 3.1. Comparison of the Longitudinal Consistency of Cyst Scores over Time

**Figure 9:**
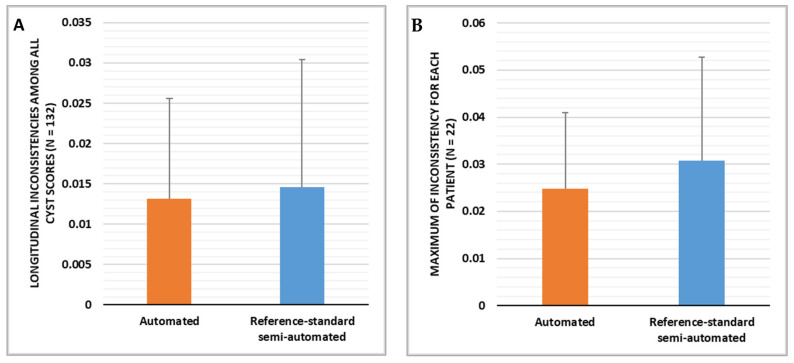
Comparison of the gold-standard semi-automated cyst segmentation and the new automated segmentation in terms of the longitudinal inconsistencies in the resulting cyst scores. (**A**) The average and standard deviation of the longitudinal inconsistency measures from all CT scans. The two methods had statistically equivalent levels of longitudinal inconsistency (*p*-value = 0.41). (**B**) Statistics on the maximum longitudinal inconsistency for each patient. The average of the maximum values from the 22 patients is graphed, with error bars representing the standard deviation among the patients. The two methods had statistically equivalent levels of maximum longitudinal inconsistency for individual patients (*p*-value = 0.21).

The first result in this category included measures of longitudinal inconsistency from every group of three consecutive cyst scores in every patient that had three or more CT scans in the study period. There were 132 measures from 22 patients. The longitudinal inconsistency of the reference standard semi-automated method was 0.013 ± 0.015 expressed in mean ± standard deviation, and the longitudinal inconsistency of the automated method was 0.012 ± 0.016 (Figure 9A). The two were statistically equivalent, with a *p*-value of 0.41.

The next result in this category looked at the worst-case inconsistency for each patient based on the maximum longitudinal inconsistency within the time course of that patient. With the reference standard semi-automated method, the average maximum longitudinal inconsistency among the 22 patients was 0.025 ± 0.031 (mean ± standard deviation), and the average maximum longitudinal inconsistency of the automated method was slightly lower at 0.016 ± 0.022 (Figure 9B). The difference did not reach statistical significance (*p*-value = 0.21).

### 3.2. Comparison of the Correlation between Cyst Scores and Pulmonary Function Test Results

**Figure 10:**
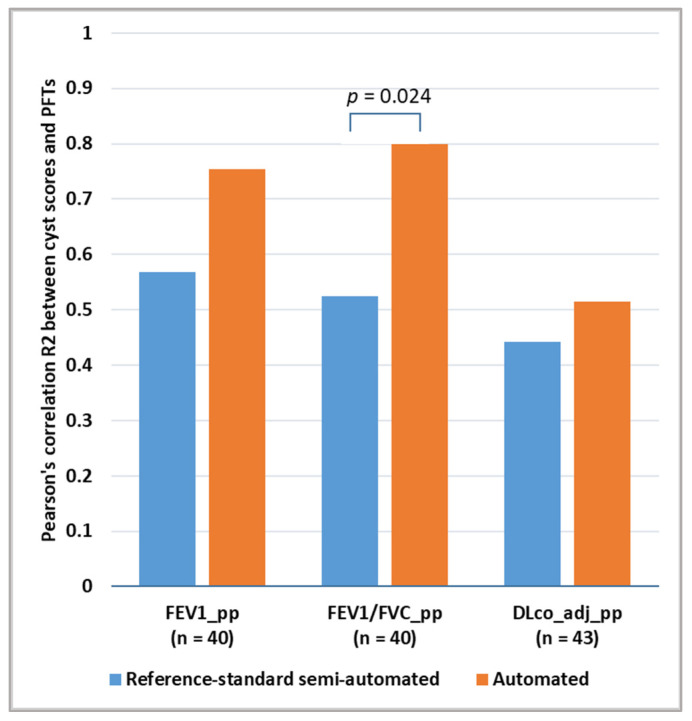
Comparison of the gold-standard semi-automated cyst segmentation and the new automated segmentation in terms of the correlation between the resulting cyst scores and the pulmonary function test results. The R^2^ values of Pearson’s correlation are graphed. The automated method provided cyst scores that were more strongly correlated with the pulmonary function results. The difference was statistically significant for the correlation to the spirometry test of FEV1/FVC (% predicted), with a *p*-value of 0.024.

A total of 43 CT examinations from 10 patients had concurrent pulmonary function tests performed during the same hospital visit. These scans were included in the calculation of Pearson’s correlation between the cyst scores and the pulmonary function test results. For the gold-standard semi-automated method, the R^2^ values of the correlation between the cyst score and the spirometry test results were 0.568 for FEV1_pp and 0.525 for FEV1/FVC_pp. The correlation between the cyst score and the diffusion capacity DLco_adj_pp was 0.443. For the automated method, the R^2^ values of the correlation between the cyst score and the PFT results were higher, with an R^2^ value of 0.754 for FEV1_pp, 0.799 for FEV1/FVC_pp and 0.515 for DLco_adj_pp (Figure 10). The difference in the correlation with the PFT result of FEV1/FVC_pp was statistically significant at a *p*-value of 0.024. The difference in the correlation with the other two PFT results did not reach statistical significance (*p*-values of 0.14 and 0.65).

## 4. Discussion

Lymphangioleiomyomatosis is a genetic disease that manifests in the lungs as the progressive formation of air-filled cysts that replace normal parenchyma. In the relatively long course of the disease, accurately monitoring the extent of cystic changes in the lungs by computed tomographic scans is an integral part of the management of the disease, with implications for treatment decisions such as the use of medication and consideration for lung transplantation [12]. Given the length of time involved, the quality and characteristics of the CT images can vary widely over time with the changes in scanner models, technologies, and protocols, making it challenging to obtain consistent and accurate measurements of the cystic damage in the lungs. The current gold-standard semi-automated analysis depends on the input of trained operators to overcome the variability, but it is susceptible to inter- and intra-operator variabilities and can be impractical for longitudinal studies covering periods of several decades.

To meet the need for large-scale studies involving diverse imaging conditions, we developed a method of fully automated cyst segmentation. A key element of the method was to derive local radiodensity thresholds for cysts from the images themselves, using information from the large airways and applying the graphics processing tool of local smoothed histogram filtering. We evaluated its performance against the current gold-standard semi-automated method in a retrospective study of 192 CT scans from 12 scanner models over a period of 23 years. We looked at a common measure of the extent of cystic changes in the lungs called the cyst score, which is defined as the total volume of cysts as a percentage of the total lung volume. The quality of cyst segmentation was assessed by two metrics: the inconsistency (rapid fluctuations) of the cyst score over time for individual patients and the correlation of the cyst score with pulmonary function test results from the physiology laboratory. The first metric was a new metric that is relevant to monitoring disease progression over time in individual patients. The second metric was used extensively in previous studies of pulmonary cysts in LAM [10,11,12,13,14,15,16,17,18].

The results showed that the automated method provided the same longitudinal consistency as the gold-standard method both in terms of the average fluctuations (0.012 vs. 0.013, *p*-value = 0.41) and the maximum fluctuation in each patient (0.016 vs. 0.025, *p*-value = 0.21). The automated method provided cyst scores that were more strongly correlated with the pulmonary function test results than the gold-standard method, particularly in the correlation with the spirometry result of FEV1/FVC (*p*-value of difference = 0.024).

The results suggest that the automated cyst segmentation had the same longitudinal consistency as the gold-standard semi-automated method. On the other hand, it was more accurate than the gold-standard method in terms of the correlation of the cyst scores with pulmonary function tests, even though the operators of the standard method were experienced in cyst-scoring LAM patients. A limitation of this work is the number of CT scans that had concurrent pulmonary function tests (n = 43), which limited the statistical power when comparing the correlations between cyst scores and pulmonary function results. Another limitation is that the automated method is not yet approved for standard patient care and therefore can only be used for research studies and not for routine clinical reports.

The automated method was implemented on a personal workstation with the data language IDL (NV5 Geospatial, Broomfield, CO, USA). The IDL language has a native layer of multi-CPU parallel processing in basic array and matrix calculations, although the computation speed was limited by the few CPU units in the personal workstation. The computation time for a typical HRCT image series of about 320 thin sections was approximately 9 min. Nevertheless, the computationally expensive elements of the method, such as the smoothed histogram filters, are amenable to large-scale parallelization on GPUs. Thus, there is the possibility of substantially accelerated computation and shortened processing times.

Combined with the advantage of being fully objective without human bias, automated cyst segmentation can be considered a competent and reliable option for saving time and labor in future clinical studies of LAM that may involve large numbers of chest CT scans from diverse scanner platforms and protocols. Apart from the measurement of the cyst score, the cyst segmentation it provides also serves as the starting point for more advanced analysis of cyst morphology and characteristics of the lung parenchyma, either near or far from the cysts, which have proven to be valuable in previous clinical studies of the nature of the disease and response to treatment [14,15,22,23,24].

## Data Availability

The data underlying the graphs will be made available for open access at https://nhlbi.figshare.com/.
